# Consciousness and its hard problems: separating the ontological from the evolutionary

**DOI:** 10.3389/fpsyg.2023.1196576

**Published:** 2023-07-07

**Authors:** Thurston Lacalli

**Affiliations:** Department of Biology, University of Victoria, Victoria, BC, Canada

**Keywords:** EM fields, theories of consciousness, the self, agency, mental causation, order from fluctuations

## Abstract

Few of the many theories devised to account for consciousness are explicit about the role they ascribe to evolution, and a significant fraction, by their silence on the subject, treat evolutionary processes as being, in effect, irrelevant. This is a problem for biological realists trying to assess the applicability of competing theories of consciousness to taxa other than our own, and across evolutionary time. Here, as an aid to investigating such questions, a consciousness “machine” is employed as conceptual device for thinking about the different ways ontology and evolution contribute to the emergence of a consciousness composed of distinguishable contents. A key issue is the nature of the evolutionary innovations required for any kind of consciousness to exist, specifically whether this is due to the underappreciated properties of electromagnetic (EM) field effects, as in neurophysical theories, or, for theories where there is no such requirement, including computational and some higher-order theories (here, as a class, algorithmic theories), neural connectivity and the pattern of information flow that connectivity encodes are considered a sufficient explanation for consciousness. In addition, for consciousness to evolve in a non-random way, there must be a link between emerging consciousness and behavior. For the neurophysical case, an EM field-based scenario shows that distinct contents can be produced in the absence of an ability to consciously control action, i.e., without agency. This begs the question of how agency is acquired, which from this analysis would appear to be less of an evolutionary question than a developmental one. Recasting the problem in developmental terms highlights the importance of real-time feedback mechanisms for transferring agency from evolution to the individual, the implication being, for a significant subset of theories, that agency requires a learning process repeated once in each generation. For that subset of theories the question of how an evolved consciousness can exist will then have two components, of accounting for conscious experience as a phenomenon on the one hand, and agency on the other. This reduces one large problem to two, simplifying the task of investigation and providing what may prove an easier route toward their solution.

## 1. Introduction

There is no shortage of theories as to the nature and origin of consciousness (Atkinson et al., 2000; Van Gulick, 2018; Seth and Bayne, 2022). Few, however, whether philosophical, psychological or computational in their focus and assumptions, explore the role played by evolution in a thorough and systematic way. Yet evolution is an essential component of explaining biological innovations of any kind, in that, as expressed by Dobzhansky (1973), “nothing makes sense in biology except in light of evolution.” Perhaps consciousness will prove to be unique in this respect, and ultimately explainable without reference to evolutionary processes (Rosenthal, 2008), but there is good reason, based on past experience, to doubt this until it can be convincingly demonstrated. This is certainly the case for anyone adopting biological realism as a stance (Revonsuo, 2018), because any comprehensive theory must address the problem of how consciousness will have changed over time. This makes a consideration of evolution unavoidable, especially so for those interested in the distribution of consciousness in taxa other than our own, a topic currently attracting increasing attention (Fabbro et al., 2015; Irwin, 2020).

It would be easier to assess the claims of competing theories of consciousness, as to what they do and do not require of evolution and evolutionary processes, if we had a conceptual framework that could be applied across theories. To this end, and to provide a point of reference for the analysis that follows, I introduce here a consciousness machine that can be reconfigured to accommodate different categories of theory. I begin by considering its applicability to what I will refer to as neurophysical theories, defined here as those where innovation at the neurocircuitry level has enabled neurons to manipulate some aspect of physical reality so as to produce conscious sensations. This is equivalent to neuroscientific stance (Winters, 2021) and dependence on some aspect of “the physical” (Godfrey-Smith, 2019), generally attributed to the action of electromagnetic (EM) fields and the like (Kitchener and Hales, 2022). There are various arguments to be made as to why, in principle, EM field theory should be central to any explanation of consciousness (e.g., see Hales and Ericson, 2022), but my intent in this paper is a more limited one, of illustrating the utility of a neurophysical stance when it comes to thinking about the evolutionary origins of consciousness. The alternative, of adopting a non-neurophysical stance, means attributing consciousness to the connectivity of neural circuits in and of itself, irrespective of any physical consequences of activating those circuits beyond the processes their connectivity sets in motion. This would include computational theories of diverse kinds (Sun and Franklin, 2007; Stinson, 2018) along with those classed as process-based, substrate-independent or functionalist (Atkinson et al., 2000; Levin, 2023), including higher-order and other representational theories (Gennaro, 2018; Lycan, 2019). However, since the source of phenomenal experience is not always specified in higher-order theories, proponents of the same theory can differ on whether or not neurophysical inputs are required at the phenomenal level (e.g., see Gennaro, 2018 on representational theories). This complicates the task of assessing those theories from an evolutionary perspective, where accounting for the emergence of subjective experience of any kind is a central concern (Feinberg, 2023), meaning any manifestation of what philosophers would call a first-person perspective, or in other contexts sentience, subjectivity, or phenomenal (or P-) consciousness. Questions relating to the neurophysical basis of higher order functions such as binding (Revonsuo and Newman, 1999; Feldman, 2012), or for solving the combination problem (Hunt and Schooler, 2019), are separate concerns and beyond the scope of this account.

In contrast with the neurophysical stance, theories or variants of theory that either reject neurophysical explanations for phenomenal experience or are agnostic on the issue will be grouped together as algorithmic theories. This necessarily means lumping together theories that are otherwise quite different, and to be clear, the term algorithmic is applied here in its most general sense, to refer to any sequence of events that achieves an end through actions that follow a predetermined set of rules or constraints. Patterns of synaptic connectivity are, by this measure, sufficient constraints, so they function in an algorithmic way irrespective of the formal similarities they may or may not share with computer programs and mathematical procedures. Further, wherever dynamic features such as synaptic plasticity are required, this can be accommodated by having a suitably constructed set of rules. To paraphrase Kitchener and Hales (2022), algorithmic theories in their purest form (here, fully algorithmic theories) rest on the proposition the connectome provides a sufficient explanation for consciousness in all its aspects where, for a neurobiological system, we are freed from the limitations of treating the connectome as a rigidly engineered structure incapable of real-time change.

Algorithmic processes as broadly defined are of course widespread in non-conscious neural events as well as conscious ones. The reason for choosing the term in this instance is specifically to emphasize an evolutionary point: that from an evolutionary perspective, the crucial difference between theories of consciousness has less do to with different ways they explain the higher-order functions of a fully evolved consciousness like our own, than their position on the nature of the neurocircuitry innovations that produced the simplest of phenomenal contents in the first instance. Here there are only two possibilities: that these innovations depend on neurons evolving novel ways to manipulate physical reality at the EM field level, i.e., the neurophysical option, or not. If not, then by default the contribution those innovations make to emerging consciousness can only be explained in terms of what algorithmic processes are capable of accomplishing in and of themselves.

An issue that emerges as especially important in the analysis that follows is that of agency, meaning, for the individual, the ability to consciously initiate and control behaviors. If we think of this in terms of the top-down control of voluntary action, then it is indeed a complex issue (Morsella, 2005; Morsella et al., 2020). An evolutionary approach is simpler in focusing attention first and foremost on explaining, from a scientific standpoint, how subjective experiences can be more than just byproducts of neural activity, epiphenomena in other words, that exert no controlling effect over behavior. This question is explored at some length, and leads to a consideration of the concept of a “self” endowed, among other attributes, with agency. The self concept is widely discussed in the literature (e.g., Panksepp, 1998; Damasio, 1999; Feinberg, 2011; Marchetti, 2012; Merker, 2013; Peacocke, 2015), and has proven a useful device, both to account for agency and other higher-order functions. Examining agency from an evolutionary perspective, and specifically how it originates, then leads me to a reconsideration of the hard problems as seen from that perspective.

## 2. A neurophysical consciousness machine

**Figure 1:**
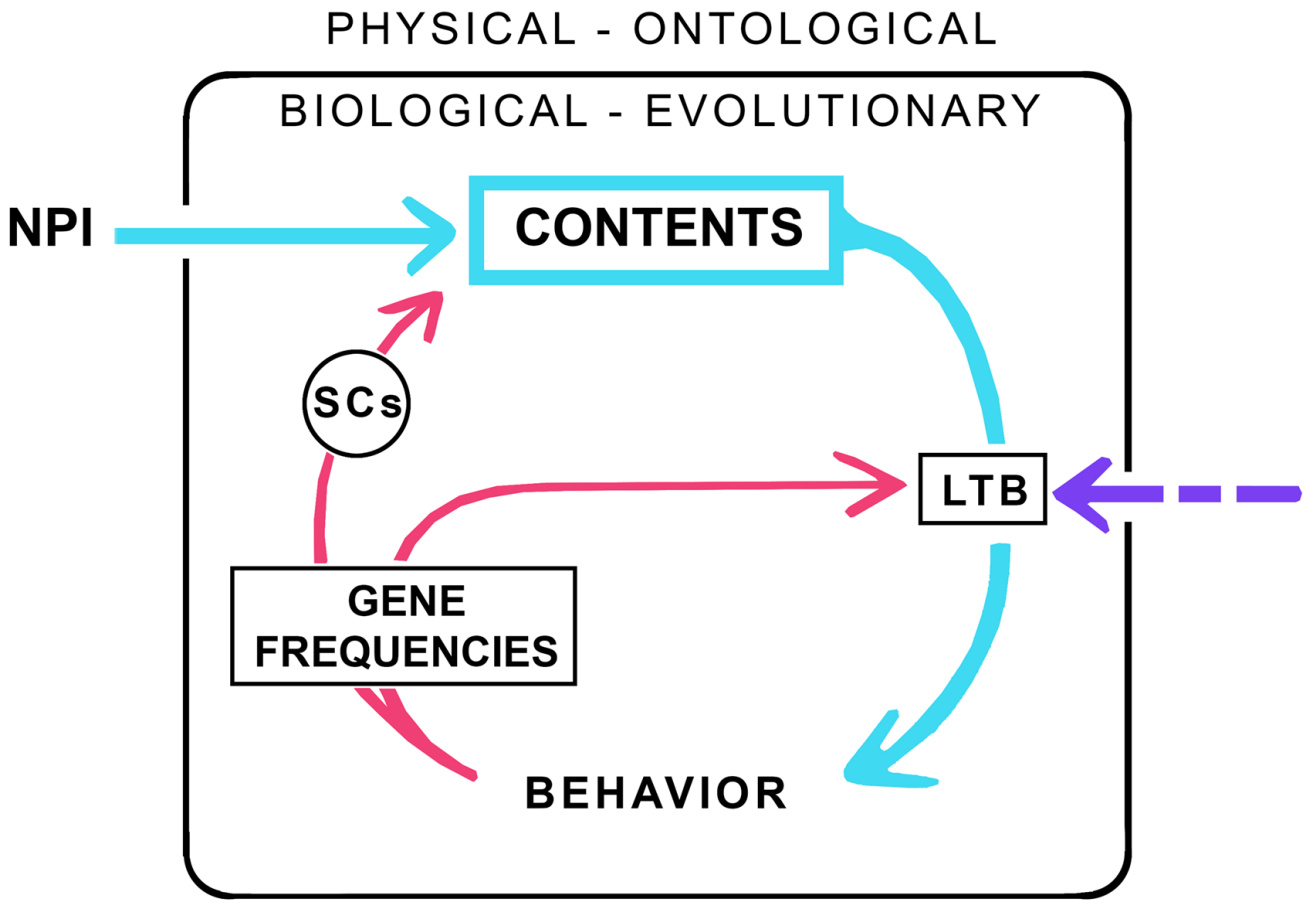
A consciousness machine configured, in this example, as the minimum required for consciousness to evolve given a neurophysical input (NPI). The internal workings of the machine, comprising the evolving neural structures and circuitry that make consciousness possible, are separated from the ontological realm, of physical rules and constraints on which life depends, where the input in question is that subcomponent of physical influences specifically required for consciousness to emerge from an otherwise non-conscious brain. How the emerging contents of consciousness are then elaborated and refined depends on natural selection, with each cycle (each turn of the “wheel”) moving the system, meaning the breeding population as a whole, through one generation. The descending half of the cycle (arrows on the right, in blue for conscious neural pathways) represents the effects of emerging contents on behavior, while the ascending half of the cycle (red arrows on the left) represents the effects on brain structure and circuitry in the next generation due to the differential effect of emerging consciousness on survival and reproductive success. To complete the cycle, it is essential that there be a link between emerging consciousness and behavior (the arrow labeled LTB, the link to behavior), but the nature of this link, whether simple or complex, or endowed with agency or not, is not specified. The LTB may itself depend on an external input from the ontological realm as indicated by the dashed purple arrow, or it may not. If the latter, meaning that the LTB is entirely algorithmic in nature, the dashed arrow would vanish. Specific neurocircuitry features are not shown except for the selector circuits (SCs). SCs are the subset of neural correlates of consciousness (NCCs) responsible for selecting a given category of experience rather than some other, meaning they exert a direct causal influence on the nature of the experience that is evoked by a given stimulus. And, since the machine itself is an evolving system, its internal mechanisms will change over time, as will the contents which, if simple to begin with, will become increasingly complex.

My consciousness machine (Figure 1) has a large wheel, much like an old-fashioned coffee grinder, which when turned through successive cycles, grinds out contents. The casing enclosing the machine separates the workings within, of biology and evolution, from the external ontological realm and the physical laws governing the universe as a whole. Biology of course depends on those laws, and on the material world more generally, but the intent here is to single out the specific inputs required to support consciousness in individual brains beyond what is required of the physical realm by those same brains to function without consciousness. And, because the machine is intended to model evolution, it operates at a population level. Hence the box labeled CONTENTS represents the mean and variance of the contents of consciousness measured across the population, and likewise for the other components of the machine. Each turn of the wheel then marks the transition from one generation to the next, with the descending pathway on the right representing effects of emerging and evolving contents on behavior, which then, via effects on survival and reproduction, alter gene frequencies in the next generation, brain circuitry, and the conscious contents those brains produce. The figure is schematic and agnostic about the nature of the neurocircuits involved, whether localized or spread diffusely across larger cortical networks, nor should it be taken to imply that functions shown as formally separate need necessarily be carried out by separate groups of neurons rather than a single group, or even a single neuron. One category of circuits is singled out: the selector circuits (SCs, see Lacalli, 2021), equivalent to the differences makers of consciousness (DMCs) of Klein et al. (2020). These are the subset of neural correlates of consciousness (NCCs) responsible for selecting a particular kind of subjective experience rather than some other, and so, in one form or another, are an essential feature of any explanation for consciousness that depends on neurocircuitry.

I begin by considering a machine configured as a neurophysical device. I do this not because of a preference for neurophysical theories over the alternatives, but because the constraints imposed by physics on neurophysical theories limits the range of competing models and ideas that need to be considered in comparison with theories for which there are no such constraints. Neurophysical theories then have a significant advantage in terms of their practical utility for exercises of this kind. What distinguishes the neurophysical machine from all others is that there will be an input from the physical/ontological realm (NPI in the figure) where, as above, the input is whatever is specifically required to support conscious brain functions over and above the physical requirements for brains to function without consciousness. What this input might be is a matter of conjecture, but most proponents of neurophysical theories assume it involves as yet inadequately understood electromagnetic field effects (McFadden, 2020; Hales and Ericson, 2022; Kitchener and Hales, 2022), though something more exotic, perhaps at the quantum level, could also play a role (Tegmark, 2015). However, since nothing specific is known about how consciousness is to be accounted for using a field-based explanation, adopting a neurophysical stance means asking more of physics than it is currently able to deliver. As Block (2009) has framed the argument, such an explanation would require a conceptual leap beyond what is currently known, which collectively puts us in the position of someone trying to explain lightning in the thirteenth century. Equating this to a hard problem means that “hard” in this usage is not a claim that the problem is uniquely intractable, only that the nature of the solution is not evident at this point in time.

For an evolving consciousness, the contents of consciousness (CONTENTS in the figure) will change over time, and where this involves an increase in complexity, the expectation is that the internal working of the machine, i.e., the neural circuitry on which these changes depend, will become correspondingly more complex. However, for any of this to happen, there must be link between the emerging contents of consciousness and behavior (LTB in the figure, the link to behavior), as there is otherwise no route by which those contents can be changed in a non-random way in consequence of natural selection. As to how this link arises, there are two possibilities. First, it may be of neurophysical origin so that, as with NPI, it ultimately depends on an external input (the dashed purple arrow). Or, it may be entirely algorithmic, meaning no such input is required (the dashed arrow would vanish). Hence, even if we defer to physics on the question of ultimate origins and the nature of the NPI, there remains the problem of accounting for the link to behavior. Much of the remainder of this account is designed to address this issue.

## 3. Evolutionary process: emergence and bootstrapping

So, where does the LTB come from? Consider first the question of how anything novel arises in evolution. The answer is that it emerges by the selective amplification of random variations at the genetic level. But selective amplification can occur during development as well, allowing neural structures and their connectivity to be reordered by real time kinetic processes as the brain develops. The Turing mechanism used to explain pattern formation during embryogenesis provides a model for how this might occur, and though there have been specific proposals for how the mechanism might apply to networks of interacting neurons (e.g., Schmitt et al., 2022), my interest here is in the more general principle involved, of the extraction of order from fluctuations across timescales (Lacalli, 2020, 2022a). The reference to timescales here is a recognition that ordering in evolving biological systems can occur both during development, in real time, and across generations due to genomic innovations encoded at the molecular level. Investigating phenomena that combine development and evolution together thus introduces an unavoidable complication, of having to deal simultaneously with two incompatible timescales, an issue discussed more fully below. But here, as a first step, I want to illustrate the utility of the order-from-fluctuations principle as a conceptual device for understanding emergence both in general terms and as it relates to consciousness.

Order, in this context, can be thought of as arising though selective amplification of random fluctuations inherent in the constituent structures and dynamics of a less ordered starting point. Consider first a situation where this involves a real-time process of synaptic reordering that occurs during brain development as a consequence of Turing-type competition. Other mechanisms could clearly be involved, as there are a multitude of other ways to produce spatial and structural order during development. But of all these options, Turing’s is arguably the most useful from a heuristic standpoint in having analytical solutions, so the underlying principles on which it depends can be understood in mathematical terms. The other point to emphasize is that, though Turing’s model can generate order (i.e., pattern) from a disordered (unpatterned) starting point, its more useful feature in broader developmental terms, and for brain development in particular, is its ability to drive processes already producing an ordered outcome toward a specified subset of all possible ordered states. In other words, the resulting pattern, whether of digits on a limb or synaptic arrays on a set of dendrites, will be ordered in a particular way rather than any other. What is then required to produce a circuit capable of a rudimentary form of consciousness by this means is for the starting point to involve a category of neural circuits sufficiently close to having the capability of producing some form of subjective experience, that random variants in that circuitry can produce a rudiment of that experience of a size suitable for further amplification. In that sense, the system must already be “on the cusp” of evolving consciousness. For a neurophysical theory this would mean that a category of circuits is present that already have at least some of the capabilities required for subjective experience to be extracted from the neurophysical source on which that experience depends. Such circuits need not necessarily be complex, but greater complexity has the advantage providing more raw material for evolution than would be present in simpler brains. For algorithmic theories, in contrast, we require the presence of circuits specifying an algorithmic process that is in some sense on the cusp of producing a conscious state. This could, for example, involve an emergent self as discussed below, but the important point is that the order-from-fluctuations principle can be applied across theories. Hence, irrespective of the theory one adopts, the answer to the question “where did it come from?” applied to consciousness, is that it was already there in a rudimentary form, hidden in the fluctuations, meaning circuitry variants that randomly arise from a genomic or developmental source. But then, because a starting point is required that is already on the cusp of making the transition to consciousness, the real puzzle is moved back a step to the preconditions necessary for the system to be on that particular cusp.

The same conceptual framework can also be applied to the link to behavior, whether this has a neurophysical source or is entirely of algorithmic origin. But there is a further problem, that without a link to behavior evolution has no way of selectively amplifying anything. In my previous analysis of emergence using Turing’s model (Lacalli, 2020), I chose to assume the link was present, and with that as a precondition, circuits capable of generating conscious contents could in principle emerge from the preconscious condition. What was missing was a consideration of how it is possible for conscious experience to be amplified from fluctuations when the link to behavior is itself just emerging by selective amplification of fluctuations in circuitry capable of producing that link. In other words, for the first conscious contents and the link to behavior to emerge together they must each, in effect, bootstrap the other at every step along the way. Precisely how this might occur is less important than whether in principle it can, which would require that the system be on two cusps at once, of producing both an emergent conscious experience and a link to behavior. The question of which came first does not arise because, much like the chicken and egg conundrum, the evolutionary answer is entirely straightforward: that neither can come first when both are equally essential at every step.

This account would not be complete without a further remark on innovation at the genomic level. Changes in the genome alter the developmental program and the way it is implemented, producing highly ordered structures in many cases without the intervention of global, dynamic patterning mechanisms like Turing’s. I have referred to this non-global, more case-specific mode of control over developmental events as programmatic assembly (Lacalli, 2022a), but it shares with the Turing mechanism a dependence on energy dissipation and irreversible thermodynamics. And in both cases order arises through amplification of random variation inherent to the system, but in different timescales. This is because a dynamic mechanism like Turing’s can reorder developmental outcomes in real time, whereas the ability of programmatic assembly to achieve a deterministic error-free result in real time depends on the way the genome has been reordered in the past, i.e., in evolutionary time, from generation to generation. This would apply as well to other rules-based patterning mechanisms, including cellular automata, where specific rules are applied in an iterative way (for examples see Berto and Tagliabue, 2022). It is premature to judge whether this latter mechanism, Turing’s, or any other dominates in the assembly of the neural circuits responsible for consciousness, but for my purposes this does not matter when it is the underlying principle, of order from fluctuations, that is the primary concern.

## 4. Evolving a minimal behavioral link: a neurophysical scenario

**Figure 2:**
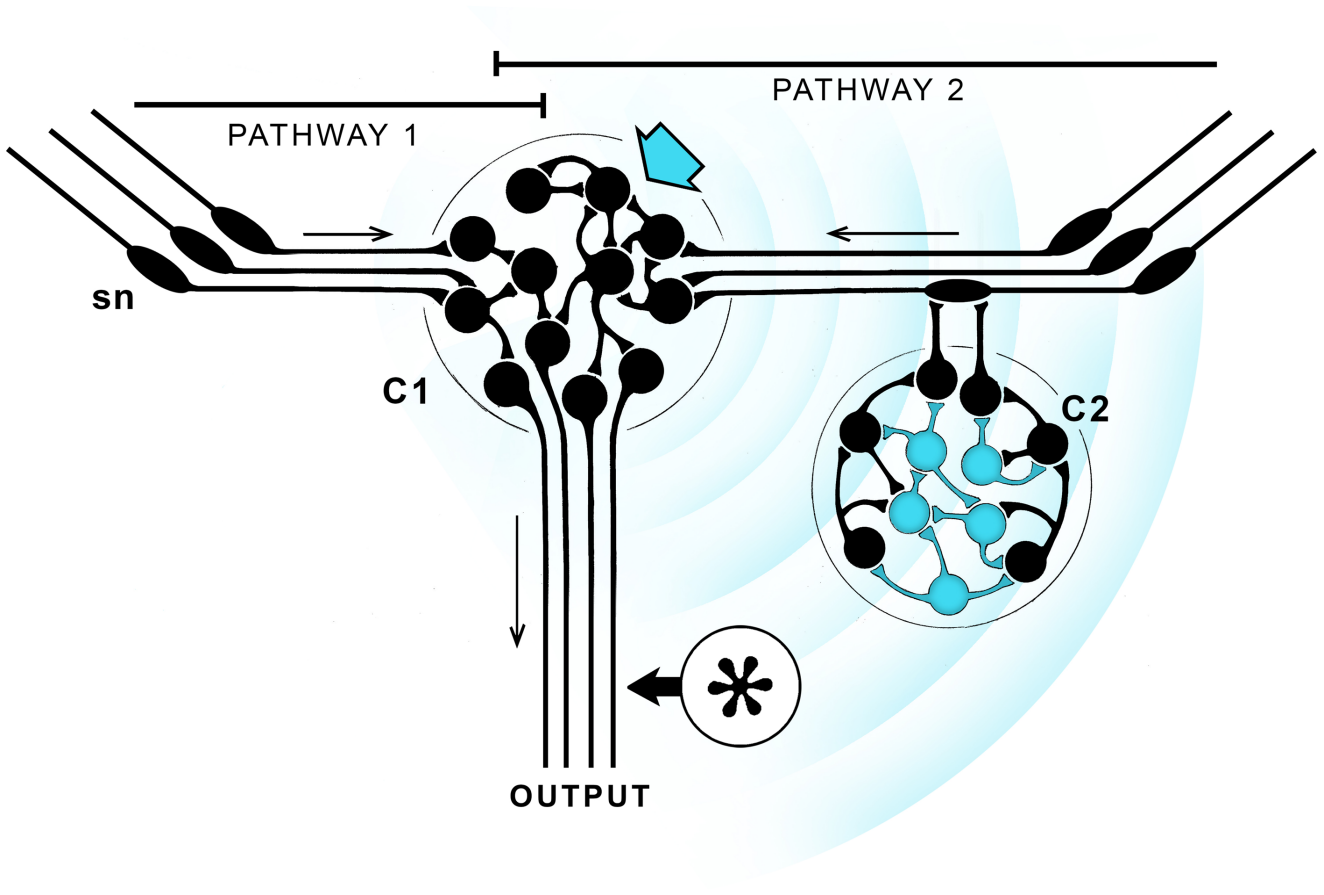
Avoiding the epiphenomenal trap: how a link to behavior might evolve given neurophysical assumptions, that consciousness depends on a EM field effects that can propagate across 3D space. Since the fields are supposed here to play a role in both generating contents and affecting behavior, this example would correspond to the workings of a consciousness machine, as in Figure 1, with external inputs to both emerging contents and the LTB. The starting point for this thought experiment is an integrative center (C1) with redundant sensory input (sensory neurons, sn, are shown with projecting cilia, and the direction of transmission by arrows) via two pathways that are assumed, in the absence of any effects ascribed to consciousness, to be functionally equivalent. Pathway 2 then differs from pathway 1 in incorporating a cluster of neurons (blue arrow) able to produce EM field effects capable of being consciously perceived that propagate (concentric blue arrow) and preferentially affect a separate subset of neurons (in blue) belonging to a second integrative center, C2. In fact the functions ascribed to C1 and C2 could be combined in a single center so the distances involved would be much reduced, but for purposes of illustration it is easier to separate them. C2 could then in principle act upstream of C1, as shown, or downstream (at the asterisk), without altering the argument. Suppose then that sensory inputs to pathway 2 can, under suitable conditions, generate a field effect that modulates C2 input, thereby altering the combined output of both pathways by changing the balance between them. If pathway 2, operating in conscious mode, produced a more adaptive outcome than pathway 1 acting alone, the optimal balance between the two would be one favoring pathway 2, which would then be strengthened generation by generation. This could involve adjustments to the character of the signal, making its dominant components an optimally selected subset of all possible EM field effects. A conscious experience of a specific kind will then have evolved, but the decisions made in consequence of this process will have been made by evolution acting over a series of generations, not by the individual in real time. The result, which applies to all such schemes so far as I can determine, is a form of consciousness without agency, where the individual lacks the ability to consciously control its own behavior in real time.

This section examines a scenario devised to account for how a link to behavior might first have evolved, not to argue the case, but to clarify some key issues. Theoretical stance matters, and since neurophysical theories are better constrained by physical principles than the alternatives, I will cast the argument in terms of EM field effects. The premise then is that the brain in question contains neurons able to generate a suitably configured EM field capable of being consciously perceived where, for the latter function, we require a subset of neurons that are differentially responsive in order to ensure the response is specific to that subset of neurons, as opposed to being subsumed in the background of electromagnetic field effects to which all neurons respond. Figure 2 shows one way of satisfying those conditions. The starting point is a pair of sensorimotor pathways, one of which (pathway 2) is modulated by inputs from an integrative center (C2) responsive to a particular subset of EM effects generated by the central integrative center (C1) where this subset of effects have the potential to be consciously perceived. A further assumption is that the outputs from the two pathways are identical in the absence of such input, which means pathways 1 and 2 will differ in their output only when EM effects of a specified kind, i.e., those capable of being consciously perceived, are present. Should the situation then arise where the “conscious” pathway, i.e., pathway 2, is more adaptive, that pathway will be strengthened over a series of generations at the expense of pathway 1, which will be suppressed or lost. The proximate reason for this outcome might be any number of things, say, that modulation via consciously perceived EM field effects produces a slight delay in activating a motor response in the presence of a particular olfactory stimulus, or sped up that same response, in either case to the benefit of the individual. The result either way is to produce a neural pathway modulated by signals capable of generating a conscious experience.

Because Figure 2 is highly schematic, some further remarks required to avoid misunderstandings, chiefly as to how EM effects act across distance in nervous tissue. The broadcast signal is shown in the figure as a wave propagated from C1 across empty space, but the intervening space would in fact be packed with neurons and nerve fibers, each capable of generating local field potentials in its own right. It is then the resulting coupling between neurons (ephaptic coupling, Weiss and Faber, 2010; Anastassiou and Koch, 2015; see also Supplement A to Hales and Ericson, 2022) that would propagate the signal, which also means the character of the signal can change with distance in ways that would not be possible for waves propagated in a passive medium. The point that then needs addressing is what an emergent experience would be like in such a situation and how it would evolve. Consider first that the signal at any point in the space can be thought of as being composed of different waveforms that each differ in their effect on the neurons responding to the signal. Assuming some waveforms activate the circuit in a more optimal way than others, the neuronal structures and configurations that generate those waveforms will be selected and enhanced over structures and configurations that generate less optimal waveforms. This will change the character of the signal which, as the broadcast center (C1) evolves, will be refined and optimized, while the response capabilities of neurons in C2 are likewise optimized. The waveform and the conscious sensation it generates will change accordingly, but the consequences of this at a behavioral level are due solely to the changing balance in output between the conscious and non-conscious pathways across generations. The key point here is that all of this happens without reference to the way the resulting sensation is actually experienced by the individual, the reason being that Figure 2 provides no route by which the character of an experience can be monitored by that individual. Nor is there a way to alter behavior in real time, because the balance between pathways, and hence behavior, only changes on an evolutionary timescale, across generations. In consequence the individual lacks agency, meaning the ability to initiate and terminate actions consciously under its own volition in real time. The sensations generated by activating pathway 2 then need not correspond to any of those experienced by animals with agency, such as ourselves, because the subjective character of the experience is irrelevant.

Figure 2 includes a second option, where C2 is moved (to the asterisk) so it directly modulates the output pathway. The field effects would then act on an integrative center able to influence motor output directly. But the result is the same, that in both cases what is happening is that evolution is adjusting the balance between the purely reflexive component of the circuit and its conscious counterpart so as to optimize that balance. Again, because it is evolution making the adjustment, across generations, rather than the individual acting in real time, the individual lacks agency even for behaviors that are variable, because it is evolution that determines the range of variation and the set point around which that variation occurs. And finally, though C1 and C2 are portrayed as separate, this is chiefly for ease of explanation, there being no reason that both functions could not be combined in a single a single center if, say, the spatial range of the signal was highly constrained. According to our current understanding of EM field theory this may well be the case (cf. Pockett, 2012, 2013), which tends to support the idea of multiple functions combined in a single center rather than multiple centers separated by a significant distance.

Consider now, with reference to Figure 2, what evolution has achieved by selecting pathway 2 over pathway 1. The result is a simple form of emerging consciousness that, in addition, is more than just an epiphenomenon. This is because it is now an essential component of a neural circuit that acts, when active, to alter behavioral outcomes. But neither the content of the experience nor its qualitative character play a causal role. Instead we have a behavioral switch where the “decision” as to which pathway dominates has been made by evolution. Why then, if this process bypasses the individual, involve consciousness at all? This is essentially the question posed by Velmans (2012), of why consciousness should exist if all its functions could as effectively be achieved by non-conscious circuits, with the brain operating “in the dark.” The answer is that having parallel pathways that differ enlarges the behavioral repertoire, and if a pathway that incorporates conscious experience has evolved from this starting point, then it must have provided an adaptive advantage at some point in the past, and in circumstances where the preconditions in terms of neurocircuit complexity, whatever those are, were also present. This is not a circular argument, but rather a simple restatement of the nature of evolutionary change. But, being an argument in principle, it will not satisfy those wanting a more specific, function-based explanation as to the proximate reason that consciousness first evolved.

The very fact that consciousness without agency is possible deserves some further comment. First, there are theories of consciousness that deny agency in any case, supposing it to be an illusion (Wegner, 2002; Halligan and Oakley, 2021). Experimental evidence for this view has come from work on the timing of conscious motor responses, principally by Libet (1985), though current interpretations of those results cast some doubt on his conclusions (Morsella et al., 2020; Neafsey, 2021). But the difficulty with this stance, however one interprets Libet’s data, is that consciousness and the character of its contents must then be accounted for without reference to adaptive optimization or evolutionary processes, leaving, for the biological realist, nothing of explanatory value. Hence, not surprisingly, this stance finds limited support among neuroscientists and evolutionary biologists. The second point relates to the body of behavioral studies summarized by Cabanac et al. (2009), and interpreted by them as implying an origin for vertebrate consciousness among the reptiles. If, in fact, the real obstacle to evolving consciousness such as our own is to incorporate agency, then these and similar results could be seen in a different light: that the transition across vertebrate taxa, from an apparent lack of consciousness to its presence, might instead be a transition from consciousness without agency to consciousness with agency. In consequence, there could be anamniote vertebrates swimming and crawling about today that remain at an ancestral and less evolved state, of being conscious without agency, representing in effect a stage in the evolution of consciousness frozen in time.

A final point concerning agency relates to the problem of accounting for the qualitative character of particular sensations. Cabanac (1992) has argued that a consciously perceived pleasure/displeasure axis is the key to understanding the benefits conferred by consciousness, with pleasure as the main motivator. This is a useful starting point for my argument, though with pain as my example, and specifically sharp pain, as from a pinprick. Consider why evolution would have chosen this particular sensation to motivate avoidance/withdrawal behavior, or, more to the point, why is sharp pain “painful”? The answer has two parts. First, one can ask whether there is something intrinsic to the stimulus of sharp pain that guarantees that it will necessarily be experienced in one particular way. If so, evolving a consciousness where pain is experienced as we do would be a predictable outcome. Conversely, it might be that the sensation of pain as we experience it induces an avoidance response only because evolution has ensured that it will do so, while the sensation itself has no intrinsic motivating power beyond that which evolution has assigned to it. There would then be no constraints on what sensation evolution assigns to an experience like sharp pain or any sensory experience, and what is painful could just as easily have evolved to be felt as we feel pleasure and vice versa. I raise this issue primarily to pose the question, not to answer it in a definitive way. But, as part 2 of this digression, a partial answer may be that for a restricted subset of sensory modalities the nature of the stimulus biases the choice of sensation. Consider again sharp pain, of the kind a newborn or newly hatched animal might receive by accident from contacts with sharp objects or in encounters with potential predators, and contrast this with the tactile stimulus from gentle stroking and soothing vocalizations by a parent comforting its offspring. For tactile experience as with sound, there is a frequency-dependent aspect of short vs. long wavelength components (von Békésy, 1959, 1960), where harm in this case correlates more with stimuli that are spatially more narrowly focused and hence higher pitched in the way they are experienced. This could explain the contrast in how sharp vs. soothing tactile stimuli are experienced where the bias toward higher pitch is with the former. Similarly, the association of anxiety and fear with physiological responses where time is a factor, e.g., of increased heart rate and rapid breathing, would bias any evolving sensation designed to signal those emotional states. Generalizing the argument to other sensory modalities is difficult, in part because these do not always have polar opposites requiring a binary choice. For example, for light there is an opposite condition, the absence of light, but no positive sensation signifying this absence. Likewise, though odors can be pleasant or noxious, both arise by chemical interactions of a qualitatively similar kind, implying their hedonic valence is assigned by other means. For these examples, one could suppose that the choice of a particular sensation, or quale, rather than some other, has been biased less by the nature of the stimulus than the availability of previously established conscious pathways that other modalities can draw on after the fact. So, for example, an odor signaling withdrawal would become associated in consciousness with experiences already associated with withdrawal, making valence in this case entirely independent of the intrinsic properties of the odor in question.

## 5. Behavioral links with agency

**Figure 3:**
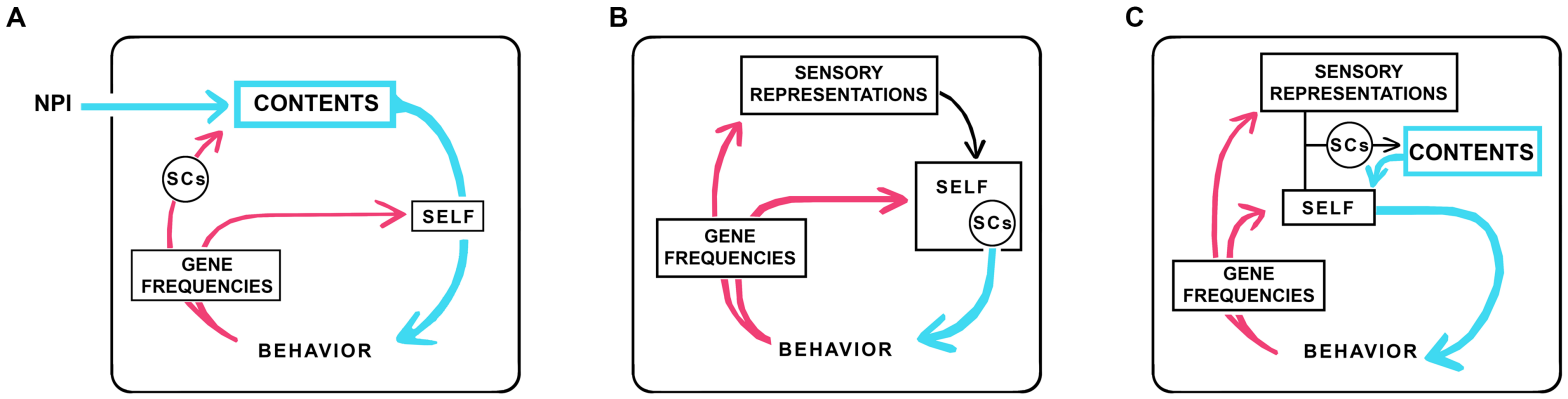
Three examples of how the consciousness machine might be reconfigured to accommodate an algorithmic self endowed with agency. **(A)** is configured for a neurophysical theory, so there is an external input as in Figure 1, but the LTB is now replaced by an algorithmic SELF. **(B)** is configured as a fully algorithmic theory with no external inputs, so the ultimate source of consciousness is algorithmic and internal to the machine. **(C)** is a more complicated version of **(B)** whose SELF is modeled on a proposal by Marchetti (2022) among others, where the self first interacts with representations of sensory processing to produce contents and, by being aware of those contents, initiates actions. There are many other ways the internal workings of the machine could be configured, the point of the figure being simply to show the formal equivalence between selves with agency across theories, regardless of whether the ultimate source of consciousness is neurophysical or algorithmic. As an aside, there is a second distinction to be made among all theories where a learning process is required for the self to acquire agency. If learning in such cases depends on a physical interaction with something external to the individual, there is in effect a physical input to the machine directed at the box labeled SELF. This is different in character from the field-dependent inputs shown in the figures, but there is a formal equivalence that begs the question of whether the learning process required for a self with agency could be accomplished in the absence of any such interaction, implying a virtual learning process where interactions with the external world were modeled using algorithms. I will defer judgment on this point, seeing no reason why evolution should opt for a virtual mechanisms given the easy access a developing brain has to sensory inputs from the real world, but it remains a question worth consideration.

The analysis above shows that, for a subset of theories, there are plausible scenarios in which consciousness could evolve without agency. How then to add agency? One approach is to think in terms of the concept of a “self.” A self is a component of numerous theories, variously conceived of as a witness and viewpoint (Merker, 2013; Williford et al., 2018), an experiencer (Cleeremans, 2011), experiencing subject (Marchetti, 2022), sentient entity (Reddy et al., 2019), or epistemic agent (Levin, 2019), but in sum, in most formulations, an entity endowed with some kind of monitoring ability, whether this is a form of awareness or something else, combined with agency. Here my concern is specifically with the self as agent (David et al., 2008) with the consciousness machine reconfigured accordingly (Figure 3). Figure 3A shows the neurophysical machine from Figure 1 with its minimal link to behavior replaced by a self with agency, while Figures 3B,C show two of many possible ways such a self-like entity might be incorporated into the machine, which could then, like Figure 3A, be neurophysical or, as in Figures 3B,C, fully algorithmic.

The first point to make about the selves in Figure 3 is that, because they are algorithmic constructs, we have no way *a priori* to place limits on what their capabilities may be supposed to be. So, for example, an emergent self could from the start be capable of converting non-conscious reflex pathways of considerable complexity directly into conscious contents. This might include somatosensory and visual maps, which would then become conscious without going through a sequence of steps where simple sensations were assembled into contents of progressively increasing complexity. However, we would still be faced with the question of how evolution assigns a particular sensation to the emergent contents, which ultimately depends on selector circuits (SCs) where the ability of each SC to evoke a particular sensation can only be systematically accounted for, regardless of theoretical stance, as a refinement achieved through an extended process of selection over multiple generations. The position SCs would likely occupy in relation to the selves in Figure 3 is: unchanged from Figure 1 in Figure 3A, as a component of the self in Figure 3B, and as part of the pathway activated by the interaction between sensory processing and the self in Figure 3C.

Now consider agency in its own right, and how it originates. To answer this in general terms we can apply the same logic used above to explore the origin of consciousness and the link to behavior for the neurophysical case. However, rather than circuits on the cusp of generating conscious experience, we must now postulate algorithmic processes on the cusp of selfness with agency. Regardless of what that entails in terms of neurocircuitry, the emerging self would then be acting simultaneously as an agent (and hence as the beginnings of a link of behavior) and as a modulator of phenomenal experience (hence its component of SCs), so the bootstrapping argument made above will again apply: that both can emerge together. There is a conceptual problem relating to the timescales involved, but I will defer this to the next section, leaving only the following difficulty: that however agency is embodied, I see no route beyond speculation to begin to answer the evolutionary question “how did it evolve?” This is because, having tried, I can state with some confidence that no amount of tinkering with scenarios like that in Figure 2 will generate a link to behavior conferring agency on the individual because, in effect, agency resides and remains throughout with evolution. Hence, in framing the question of how agency acting at the level of the individual first evolved, it is in my view more meaningful to do so, not in terms of a *de novo* origin of agency from unknown beginnings, but as a transfer of agency from evolution to the individual. This makes explicit the deeper ties that link the process as a whole, of the evolution of consciousness, with the dual nature of the timescales involved. If we then look at the recipient of agency, the individual, we are back in the realm of real-time events, and it is investigating these that is likely to prove most fruitful. The operative question is then not “how did it (agency) evolve?” but “how does it develop?” A promising approach would appear to be the one proposed by Cleeremans (2011), see also Cleeremans et al. (2020), to frame the question in terms of learning: that the brain must learn to be conscious, or in the same vein, that selfness must be learned and achieved (Marchetti, 2022). I will be more restrictive than Cleeremans, as he is concerned with higher order forms of consciousness, whereas I care only about the simplest contents, i.e., phenomenal ones. Further, my perspective is bottom-up in being concerned only with how the individual acquires agency, which prompts me to make the following conjecture: that transferring agency from evolution to the individual can only happen through the action of feedback processes operating in something other than evolutionary time, which by default means real time. Where this depends on a learning process, then memory will also be involved, so that information on the experiential result of particular actions can be stored and recalled. Establishing agency would then be inescapably an algorithmic process that operates in real time.

How broadly the above conjecture can be applied across the theoretical landscape is difficult to assess given the diversity of theories of consciousness and how little the majority of them have to say about the role of evolutionary processes. However, for theories to which the conjecture does apply, a prediction one can make is that the necessary feedback processes will occur during a phase of development when the individual is able to actively test the consequences of real-time motor activities, implicating the period from late embryogenesis through the immediate post-hatching and/or post-natal period (Delafield-Butt and Gangopadhyay, 2013; Ciaunica et al., 2021). And, if such actions are indeed obligatory for species with conscious agency, the behaviors of animals during such periods could provide an empirical test for distinguishing between species that have consciousness with agency from those that have either a simpler form of consciousness, without agency, or none at all.

## 6. Timescale-related issues: synaptic plasticity, feedback, and behavioral flexibility

**Figure 4:**
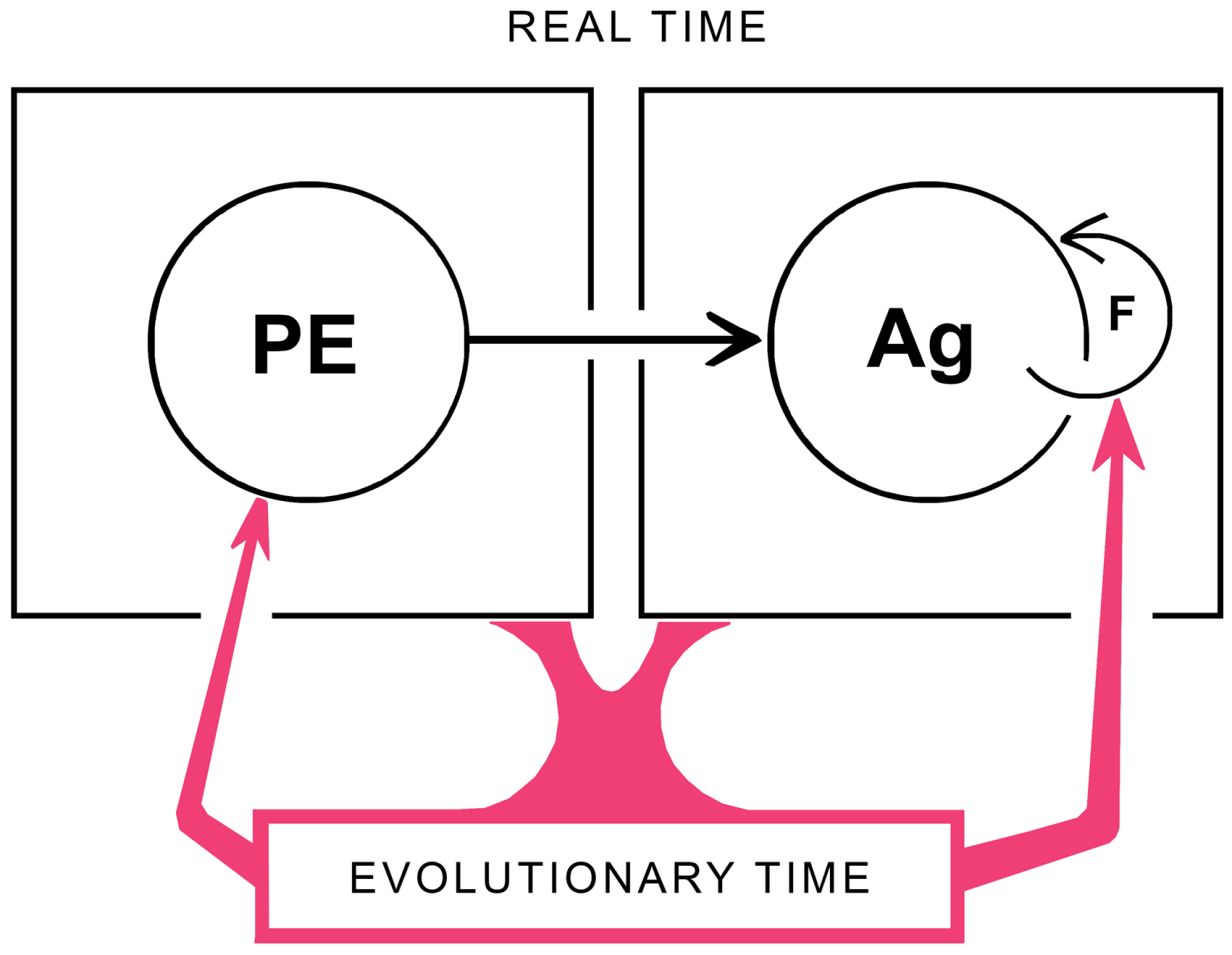
A schematic representation of how development and evolution work together to generate consciousness. Both real time (i.e., developmental) and evolutionary timescales must be included, and these are separable as shown. For the former, a key issue is that the developing brain must be capable of some form of real-time phenomenal experience (PE) in advance of the actions by which agency (Ag) can be “learned” through the real-time feedback mechanisms (F) on which that learning process depends. There are two asymmetries here that operate in real time, during brain development, that (1) Ag depends on PE but not the reverse, so the arrow connecting these is unidirectional, and (2) that Ag is adjusted and refined by real-time feedback while the subset of PEs on which this process depends operate as fixed reference points that can be altered and refined only in evolutionary time. Any conscious contents subject to alteration by late embryonic or post-natal feedback processes would then be precluded from being PEs, which would, by definition, be limited to consciously perceived sensations (i.e., qualia) that, once the neurocircuitry mechanisms required to evoke them are in place, remain subsequently unchanged by the real-time experiences of the individual. The evolutionary side of the story (in red) indicates the role genomic change plays, generation by generation, in changing both the character of PEs and the feedback mechanisms required for agency to develop. A complication is that, in evolutionary time, emergent phenomenal experience and emergent agency are co-dependent, because neither can evolve without the other. But this does not contradict the real-time asymmetry in the dependence of Ag on PEs, because for evolution, all of development is a single point in time (see text for further discussion). The key point then is that having two timescales allows phenomenal experiences and agency to evolve together while being, in effect, insulated from one another. This is an important insight in reductionist terms, justifying the separation of one large problem, of investigating consciousness as a whole, into two, of investigating phenomenal experience on the one hand and agency on the other.

Within the framework developed above, there is an important distinction to be drawn between the neurocircuitry involved in producing sensations of particular kinds, i.e., phenomenal experience, and those involved in generating agency. Logically it would appear that the former must be in place so their output can guide the learning process by which agency is established, so the period of synaptogenesis and synaptic plasticity required for the correct assembly of the circuits responsible for phenomenal experience, including SCs, would have to be over or nearly so before the learning process could begin. If restricted to embryogenesis, there would also then be no way to subsequently correct errors that occur during the assembly process for SCs without a specific mechanism in place that operates after birth or hatching in order to do so. Such postnatal mechanisms clearly operate to shape and refine complex contents, the conscious display of the visual field being a well studied example (Hensch, 2004; Levett and Hübener, 2012). For the simplest of phenomenal contents, however, meaning the qualia of experience, this appears not to be the case. In consequence, the sensations experienced by individual brains for these would be fixed at the completion of brain development however distant those sensations were from the population standard. As shown in Figure 4, this manifests as an asymmetry in the relation between phenomenal experience (PE) and agency (Ag) whereby Ag depends on PE, as indicated by the arrow between them, but not the reverse. In addition, since the only way to remove discrepancies between PEs in individual brains and the population standard is through natural selection acting over evolutionary time, feedback on the PE side of the diagram is exclusively via an evolutionary route (indicated by the red arrow on the lower left). Agency, in contrast, is like vision as a total experience in depending on feedback occurring in real time, as this is required as part of the process by which each individual adjusts its actions as it learns. Hence the feedback loop shown for agency (F in the figure) operates in real time, but has no counterpart on the PE side of the diagram. There is then an apparent contradiction with the bootstrapping argument made in previous sections, that Figure 4 incorporates the assumption that PEs develop independently of and before the learning process for Ag can begin, while bootstrapping requires PEs and Ag to mutually assist each other, implying simultaneity. The reason this is not in fact contradictory is that when the emergence of PEs, Ag and hence consciousness is being dealt with in an evolutionary context, all of development is effectively a single point in time so long as the adaptive utility of the outcome is tested only after development is complete. Hence, developmental events can be simultaneous in evolutionary time when, in real time, they are not.

Having two separate timescales is noteworthy for other reasons. First, to continue with the point made in the previous paragraph, it segregates the feedback processes required, which occur in real time for emerging agency, but in evolutionary time for the progressive diversification and refinement of phenomenal contents. For agency this is a change from the preconscious condition, and is consequential, which is why the transferral of agency from evolution to the individual has been given the emphasis it has in this account. The reason for doing so is ultimately a reductionist one, best illustrated by considering blanket statements of the form “consciousness must be learned”: that even if is correct to say that agency at the level of the individual must be learned, so that consciousness with agency also requires learning in order to exist (i.e., to evolve), there is nevertheless a good deal about phenomenal experience that can be usefully investigated without concerning oneself with learning. This is because the timescale difference insulates processes relating to the elaboration and refinement of phenomenal experience, including much of what is considered under the umbrella of the hard problem, from the mechanisms involved in generating agency. The problem of explaining consciousness is consequently reduced to two separable problems, of explaining phenomenal experience on the one hand and agency on the other, both of which can then be more easily investigated separately than they could be together. A caveat is that, because the arguments I have used here to justify this reductive step are evolutionary, it is not clear if the same result would obtain for all theories of consciousness, and specifically for theories or variants of theory that are silent on the role played by evolution. Absent an answer from my analysis, I address that question to proponents of those other theories.

A final point relating to timescales concerns the function of consciousness, where the reference here is to function in general terms rather than the specific functions consciousness may first have evolved to perform. I have addressed this previously in my analysis of selector circuits and experience space, the conclusion being that consciousness allows evolving populations to gain access to regions of both selector-circuit space and experience space that would otherwise not be available to them (Lacalli, 2021). Simply put, a greater range of behaviors is possible with consciousness than without. This is then a precise statement about function, but it should ideally be more specific about the benefits of transferring agency from evolution to the individual. Amended, a more *complete* statement is that the function of consciousness is to (1) increase the behavioral repertoire by expanding access to otherwise inaccessible regions of behavior space, and (2) reduce by orders of magnitude the time required for behavioral changes to occur in response to changing circumstance. The second point relates to what might generally be referred to as behavioral flexibility, but in an evolutionary context this term acquires a more specific meaning: of the ability of the individual to alter its behavior in real time where, absent consciousness, that same alteration could only have been achieved by natural selection operating over generational time, and hence many orders of magnitude more slowly.

## 7. Conclusions: consciousness and its hard problems

A certain amount of lumping and splitting occurs in the early stages of the development of most scientific ideas, of determining which of the various phenomena under study should be treated together in analytical terms and which should be dealt with separately. For consciousness, considered from a philosophical, psychological or neurological perspective, a considerable amount of lumping and splitting has already been accomplished, and a degree of consensus has emerged as to the issues at stake and the range of perspectives one can adopt, including the nature of the hard problems and explanatory gaps that bedevil the subject (Levine, 1983, 2009; Chalmers, 1995). Though these issues are seldom considered from an explicitly evolutionary perspective, there is an implicit evolutionary component to the analysis by Majeed (2016), who recasts what is generally considered the least tractable of the hard problems as two questions, namely (1) how subjective experience of any kind can exist in the first place, and (2) how a consciousness consisting of diverse distinguishable contents is to be accounted for. This separates the issue of origins from that of elaboration and refinement, and for theories that deal with contents as separable and individually subject to selection, elaboration and refinement are inescapably matters to be dealt with in an evolutionary context. Previous papers in this series (Lacalli, 2020, 2021, 2022b) were in fact designed to do exactly this, adding, in the current installment, an investigation of how a link to behavior that embodies agency might first have evolved. Regardless of how difficult resolving such questions proves to be, they all belong to a category that Chalmers (1995, 1996) treats as “easy” problems, as they fall within the bounds of established neuroscience and are soluble in principle using established or emerging methodologies given sufficient time (Kostić, 2017; Kitchener and Hales, 2022). In contrast, a truly hard problem would be one that tests the bounds of what science is capable of explaining, in this case how subjective experience can exist, but more precisely, how it is that something with no material existence can have definable properties of a particular type, or indeed any properties at all.

An evolutionary view recasts the issue of hard problems in several ways. First, it reinforces the supposition that the easy and hard problems referred to above should be dealt with separately. This is because, so long as the evolutionary sequence is from simple contents to more complex ones, solving Chalmers’ ultimate hard problem for a subjective experience of any kind solves it fully. Subsequent innovations in conscious experience are then a matter of elaborating neural processing in ways that conventional neuroscience should, in principle, be able to explain. Accepting that the elaboration and refinement of conscious contents is an evolutionary process also resolves the second issue raised by Majeed (his point 2, above), that if consciousness is composed of separable subcomponents, as would likely be the case for any EM field-based theory, then evolution is simply the means by which that separation is effected. But explaining consciousness as it is today in evolutionary terms raises an additional set of potentially hard problems relating, not to the limits of scientific explanation, but the fragmentary nature of the evidence available to us on unique events lodged in the distant past. Absent a relevant fossil record, we are reliant on inference to answer such questions, e.g., as to the proximate reason for which consciousness first evolved, or the sensory modality involved, and there is currently little one can say beyond speculation. The situation may improve, and would, should the relevant neurocircuitry prove to contain some form of consciousness “signature” that could be traced across taxa. But however difficult a problem this proves to be in practice, it is currently simply a matter of insufficient data, not a test of the limits of scientific explanation.

What does test those limits is the problem Chalmers identifies, now generally accepted as fundamental (Searle, 1998; Robinson, 2015), of how it is possible for any kind of subjective experience to exist. Different categories of theory will parcel out the burden of explanation in different ways. The distinction I’ve made throughout this paper is between theories that attribute the ultimate source of subjective experience to either a neurophysical cause on the one hand, or algorithmic processes on the other. To begin with the former, for neurophysical theories the burden of explanation is divided between physics, neuroscience and evolutionary biology. If we suppose that subjective experience can be conceived of as depending on EM fields composed of separable harmonic components, emergence results from the selective amplification of some of these at the expense of others. How this is done is a matter of understanding events occurring at a neurocircuitry level. Hence it belongs to the category of easy problems, and yields answers that are at best examples of weak emergence (*sensu* Bedau, 1997). The deeper problem is why the signal thus generated over a background of noise should manifest itself to the individual in a particular way, or in any way at all. This can be dealt conceptually with by assuming that subjective experience is simply a particular sum of waveforms that solve the relevant field equations, selected from many possible such sums. But knowing the waveforms does not explain why some solutions but not others should have the property of being perceived as an apparently real experience of a particular kind by a suitably configured assemblage of neurons. This is at root an ontological issue, as it concerns existence and the nature of reality. Hence it belongs in physics, even if the answer does not fit within the existing explanatory structures of physics as it is today. In this sense the very existence of subjective experience performs a useful function in alerting physicists to a significant part of reality they cannot yet fully explain. That attempts to do so have so far reached only the stage of arguments by analogy, to relational aspects of quantum behavior as an example (Smolin, 2020), shows how early we are in terms of seriously exploring the subject in scientific terms.

Fully algorithmic theories yield a problem of somewhat different kind, because subjective experience must then be conjured up out of a set of procedures carried out by a network of interacting elements where the content of that process, meaning the task it is designed to perform, generates subjective experience in and of itself. Hence physics, except perhaps physics of a radically new kind, would seem to provide little by way of assistance. Instead, we must devise *ab initio* explanations as to how a process, essentially computational in character, can give rise to something that is immaterial yet real, and exists only due to the execution of that process. This is a speculative enterprise in the extreme at this point in time, and is especially problematic for the subset of algorithmic theories that suppose the answer resides in the as yet poorly understood realm of ideas concerning the capabilities of network-based information processing. First is the problem of requiring information to be more than an epistemic convenience, in other words a way of describing reality rather than a component of that reality, is inadequate in principle as an explanation (Manson, 2010; Manzotti, 2012; Pockett, 2014; see also Kitchener and Hales, 2022 on the grounding problem). On the other hand, at a more practical level, there is the problem that the methodologies available for dealing analytically with information processing are specifically designed to be agnostic on the content of what is being processed (Wood, 2019). This is of particular concern where content is important, e.g., when considering whether a given network process has at yet achieved selfhood, or awareness, or not. My conclusion in consequence is that the supposition, that algorithmic processes in and of themselves generate conscious experiences, must be one of two things: it is either a hard problem of a very profound kind, or it is a strong argument that any fully algorithmic theory lacking a neurophysical component must be false.

## Data availability statement

The original contributions presented in the study are included in the article/supplementary material, further inquiries can be directed to the corresponding author.

## Author contributions

TL is solely responsible for the preparation and content of this article.

## Funding

This work was supported by the L. G. Harrison Research Trust.

## Conflict of interest

The author declares that the research was conducted in the absence of any commercial or financial relationships that could be construed as a potential conflict of interest.

## Publisher’s note

All claims expressed in this article are solely those of the authors and do not necessarily represent those of their affiliated organizations, or those of the publisher, the editors and the reviewers. Any product that may be evaluated in this article, or claim that may be made by its manufacturer, is not guaranteed or endorsed by the publisher.
